# Dartos pouch orchiopexy for adult testicular torsion and symptomatic retractile testis: Technique, early outcomes and critical appraisal of trans‐tunical suture fixation

**DOI:** 10.1002/bco2.70272

**Published:** 2026-08-18

**Authors:** Dinesh K. Agarwal

**Affiliations:** ^1^ Department of Surgery, Western Health University of Melbourne Melbourne Australia; ^2^ Department of Urology Royal Melbourne Hospital Melbourne Australia

**Keywords:** dartos pouch, intermittent torsion, orchiopexy, retractile testis, surgical technique, testicular torsion

## Abstract

**Objectives:**

To describe a reproducible single‐incision dartos pouch orchiopexy technique for adult patients requiring testicular fixation, report early outcomes from an initial adult series and critically appraise the evidence supporting conventional trans‐tunical suture fixation.

**Patients and Methods:**

A stepwise description is presented of a proximal‐reflection dartos pouch orchiopexy performed through a single high transverse scrotal incision, in which the parietal tunica vaginalis is reflected proximally over the spermatic cord before dependent subdartos placement of the testis without trans‐tunical or transparenchymal fixation sutures. Perioperative outcomes from an initial adult case series are reported.

**Results:**

A total of 22 dartos pouch orchiopexies were performed in 12 adult patients with a median age of 23 years (range 18–31), including eight patients with intermittent testicular torsion, two with acute torsion and viable testes following detorsion and two with symptomatic retractile testis. Structured ultrasonographic follow‐up ranged from 15 to 21 months (mean 19.2 months). Scrotal ultrasonography demonstrated preserved testicular vascularity and stable dependent positioning of all testes. There were no cases of recurrent torsion, testicular atrophy, chronic scrotal pain or scrotal complications.

**Conclusion:**

Single‐incision dartos pouch orchiopexy appears to be a safe, atraumatic and reproducible non‐parenchymal technique for adult testicular fixation and may represent a viable alternative to conventional trans‐tunical suture fixation.

## 1 INTRODUCTION

Orchiopexy is recommended for intermittent testicular torsion, viable testes following detorsion and selected cases of symptomatic retractile testis. Orchiopexy techniques for torsion are heterogeneous, with variation in incision, suture material, number of fixation points and anatomical site of fixation; however, sutured fixation remains the most commonly described approach.^1^ Such suture techniques may be associated with complications, including vascular compromise, chronic scrotal pain, testicular abscess and, rarely, recurrent torsion.^2^, ^3^, ^4^, ^5^, ^6^

Dartos pouch orchiopexy offers a non‐parenchymal alternative with favourable clinical outcomes. However, despite being recognised in the literature, detailed adult‐specific surgical illustrations remain limited.

Dartos pouch orchiopexy without placement of testicular fixation sutures was described by Redman and Barthold, who reported excellent outcomes in paediatric and adolescent patients using separate ipsilateral scrotal incisions.^7^ However, the original description provided limited operative detail, and the accompanying black‐and‐white illustrations offered limited clarity regarding the individual surgical steps. Furthermore, detailed operative illustrations of dartos pouch orchiopexy are limited in standard urological references, including Campbell‐Walsh‐Wein Urology and the Hinman Atlas of Urologic Surgery.^8^, ^9^

Building upon the dartos pouch technique described by Redman and Barthold,^7^ we describe an adult‐specific single‐incision modification incorporating bilateral access through a high transverse scrotal incision, proximal reflection of the tunica vaginalis over the spermatic cord and a detailed stepwise operative description intended to improve reproducibility and facilitate broader adoption of non‐parenchymal orchiopexy.

## 2 PATIENTS AND METHODS

### 2.1 Study design and patients

This was a retrospective review of an initial consecutive adult case series of patients aged ≥18 years who underwent single‐incision dartos pouch orchiopexy by a single surgeon between 2018 and 2024. Patients requiring orchiopexy for intermittent testicular torsion, acute torsion with a viable testis following detorsion or symptomatic retractile testis were included. Outcomes assessed included recurrent torsion, testicular atrophy, chronic scrotal pain, scrotal haematoma or infection, reoperation, ultrasonographic vascularity and dependent testicular positioning. Follow‐up included early clinical review and structured scrotal ultrasonography at 6 and approximately 18 months.

### 2.2 Surgical technique

The patient is positioned supine. A single high transverse scrotal incision is made, allowing access to both hemiscrota when bilateral orchiopexy is required. The high position of the incision also facilitates creation of dependent bilateral dartos pouches caudal to the incision. The affected testis is positioned beneath the incision while the scrotal layers are gently deepened. After opening the parietal tunica vaginalis, the testis is delivered through the incision and externalised. As the testis is delivered through the high scrotal incision, the parietal tunica vaginalis is reflected proximally over the spermatic cord.

The divergent cut edges of the reflected tunica vaginalis are approximated and secured to the superficial external spermatic fascia using interrupted 4‐0 or 5‐0 polyglactin sutures. Usually, five to six interrupted sutures are required. Care is taken to avoid deep sutures within the spermatic cord structures to prevent vascular injury or bleeding. In cases with high investment of the tunica vaginalis over the spermatic cord, any redundant tunica vaginalis that prevents satisfactory proximal reflection may be excised before the testis is placed within the dartos pouch.

A subdartos pouch is created in the plane between the dartos fascia and external spermatic fascia using Metzenbaum scissors followed by blunt digital dissection. The pouch is fashioned to comfortably accommodate the testis without compression or tension.

**FIGURE 1:**
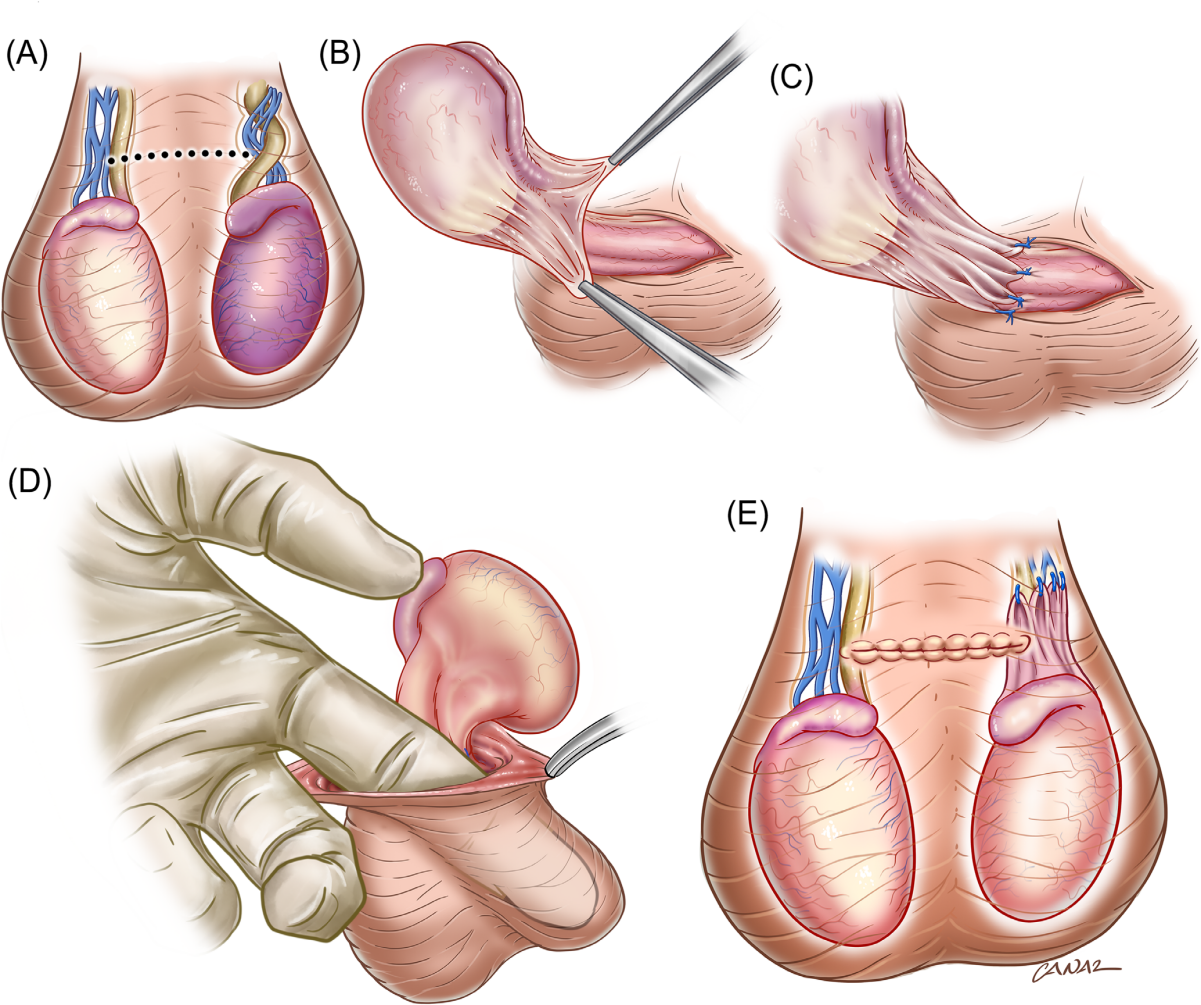
Single‐incision dartos pouch orchiopexy technique. (A) A single high transverse scrotal incision (dotted line) is made, providing access to both hemiscrota and allowing creation of dependent bilateral dartos pouches. (B) After opening the parietal tunica vaginalis, the testis is delivered through the incision. The parietal tunica vaginalis is reflected proximally over the spermatic cord. (C) The divergent edges of the reflected tunica vaginalis are approximated and secured to the superficial external spermatic fascia using interrupted absorbable sutures. (D) A dependent subdartos pouch is created between the dartos fascia and external spermatic fascia by blunt digital dissection. The testis is then placed within the pouch. (E) Final appearance following bilateral orchiopexy through a single transverse scrotal incision. The testes are positioned dependently within their respective dartos pouches without trans‐tunical or intraparenchymal fixation sutures.

The testis is placed within the dartos pouch without placement of sutures through the tunica albuginea or testicular parenchyma. The neck of the pouch is gently narrowed above the upper pole of the testis using a 4‐0 polyglactin suture to maintain its dependent position. When contralateral orchiopexy is indicated, identical steps are performed through the same incision. The dartos layer and skin are closed separately using absorbable sutures. The key operative steps are illustrated in Figure 1.

## 3 RESULTS

A total of 22 dartos pouch orchiopexies were performed in 12 adult patients. The median age was 23 years, with a range of 18–31 years. Bilateral orchiopexy was undertaken in eight patients with intermittent testicular torsion and in two patients with acute testicular torsion following successful detorsion and confirmation of testicular viability. Two patients with symptomatic retractile testis underwent unilateral orchiopexy.

Most procedures were completed as day‐case surgeries; two patients with acute torsion required overnight admission. Postoperative scrotal discomfort was minimal. Early postoperative review within 4 weeks demonstrated well‐positioned testes within the dependent scrotal pouch without wound complications, haematoma, infection or significant pain.

After the initial postoperative visits, patients were followed with scrotal ultrasonography at 6 months and again at approximately 18 months. Structured ultrasonographic follow‐up ranged from 15 to 21 months (mean 19.2 months). Ultrasonography demonstrated preserved testicular vascularity and stable dependent positioning of all testes within the scrotum. There were no cases of recurrent torsion, testicular atrophy, scrotal haematoma, chronic scrotal pain or requirement for reoperation.

## 4 DISCUSSION

Trans‐tunical point suture fixation remains the dominant method of orchiopexy in contemporary practice. Survey data from the United Kingdom/Ireland and Europe suggest that most surgeons continue to use suture‐based fixation, and the BURST‐BAUS FIX‐IT consensus recommends suture fixation with tunica vaginalis eversion in adults while accepting both suture fixation and dartos pouch fixation in children.^10^, ^11^, ^12^ These observations highlight an important gap between prevailing practice and the limited comparative evidence supporting trans‐tunical sutured fixation as the optimal adult technique.

Experimental studies challenge the assumption that point suture fixation provides the most durable biological fixation. Morse and Hollabaugh showed that creating a window in the tunica vaginalis, allowing exposed tunica albuginea to lie against a raw scrotal surface, produced broad dense adhesions, whereas simple suturing produced only limited focal adhesions.^13^ Rodriguez and Kaplan similarly demonstrated in a rat model that point sutures produced only localised fibrosis at the suture sites, while tunica vaginalis eversion produced diffuse high‐grade scarification over a broad testicular surface. Importantly, adding absorbable or non‐absorbable sutures to tunical eversion did not improve fixation.^14^ Bellinger et al. further reported that sutured orchiopexy in rats was associated with inflammatory and histological injury, including abscess formation, tubular necrosis and impaired spermatogenesis, whereas dartos pouch fixation produced circumferential adherence with better preservation of spermatogenesis.^6^

The relevance of animal studies to human practice must be interpreted cautiously. Nevertheless, these studies provide a plausible mechanistic explanation for clinical failure after sutured fixation. The testis remains mobile within the scrotal compartment and is exposed to cremasteric activity, changes in scrotal position and repetitive traction during normal activity. A focal suture placed through the tunica albuginea and anchored to compliant dartos or septal tissue may be exposed to repetitive shear forces. Failure may therefore occur not only because an absorbable suture loses tensile strength but also because point fixation may cut through, loosen or fail to generate durable broad‐based adhesions. The key biological distinction may be focal point fixation versus broad non‐parenchymal fixation, rather than simply absorbable versus non‐absorbable suture.

Several clinical reports also challenge the assumption that sutured fixation provides permanent protection. Mor et al. reported recurrent intravaginal torsion in 8 of 179 patients operated on for unilateral testicular torsion at a single institution. Recurrence occurred 0.5–23 years after initial fixation, involved both ipsilateral and contralateral testes and occurred even after non‐absorbable polypropylene suture fixation.^2^ Similarly, von Zastrow and Sotelino identified 23 publications describing 40 patients with acute testicular torsion after previous fixation and noted six additional unpublished cases reported personally by urologists.^3^ Rodriguez and Kaplan also noted that among 13 reported recurrent torsion cases in which the original fixation technique was described, nine showed no evidence of fixation at re‐exploration, while the remaining cases had only inadequate fixation.^14^ Morse and Hollabaugh reported a case of suspected intermittent torsion after prophylactic silk fixation of the contralateral testis, where re‐exploration showed that all four silk sutures had pulled free from the tunica albuginea, leaving the testis freely mobile.^13^ These observations suggest that recurrent torsion after previous fixation is probably under‐recognised, particularly because recurrence may occur years after the index procedure and patients may present to a different institution.

Clinical studies of non‐parenchymal fixation provide supportive evidence for broad surface fixation. Lent and Stephani reported 46 tunica vaginalis eversion orchiopexies in 35 patients aged 10–30 years, with follow‐up ranging from 2 to 15 years, and observed no recurrent torsion.^15^ Redman and Barthold described 19 scrotal pouch orchiopexies in 11 boys with testicular torsion and reported no scrotal complications, testicular atrophy or recurrent torsion at a mean follow‐up of 25.6 months.^7^ More contemporary comparative paediatric studies have similarly failed to show inferiority of non‐parenchymal fixation. Koh et al. compared sutured point fixation with Jaboulay‐type dartos pouch fixation in 482 explored scrotal compartments and observed no recurrent torsion in either group at a mean follow‐up of 6.3 years.^16^ Boam et al. reviewed 585 testes left in situ following emergency paediatric scrotal exploration and found no recurrent torsion after either dartos pouch fixation or sutured fixation; however, suture‐specific complications, including pain associated with sutures, iatrogenic epididymal injury and suture sinus or granuloma, were reported in the non‐absorbable suture group.^17^

Suture‐related morbidity may also be under‐recognised. Smith and Godbole reviewed 40 boys undergoing paediatric sutured testicular fixation and reported complications in 10 patients, including haematoma, infected haematoma, wound infection, acute postoperative pain and persistent pain. One child developed stitch‐related pain requiring return to theatre for removal of fixation sutures, after which the pain resolved.^4^ In a cohort of formerly cryptorchid adult men, Coughlin et al. found that transparenchymal testicular suture was independently associated with infertility; although not torsion‐specific, this supports concern that direct testicular suturing may not be biologically innocuous.^18^ Ribeiro et al. also showed in rats that even temporary parenchymal suture penetration without tied knots produced seminiferous tubule morphological changes, with reduced sperm motility and viability in adult animals.^19^ Jarow's adult arterial‐cast study further suggests that lower pole trans‐albugineal sutures may compromise intratesticular arterial filling, providing an additional anatomical rationale for avoiding parenchymal fixation where possible.^5^ Reported complication rates may underestimate true fixation‐related morbidity because torsion is relatively uncommon, individual surgeon experience is often limited and delayed complications may present elsewhere or remain unpublished.

Moore et al. systematically reviewed orchiopexy techniques for acute testicular torsion and found marked heterogeneity in fixation methods. No included study reported ipsilateral or contralateral retorsion regardless of fixation method, but follow‐up was generally limited and the overall quality of evidence was weak. The authors concluded that available evidence was insufficient to establish superiority of any single orchiopexy technique.^1^ Taken together, current clinical evidence does not demonstrate that non‐parenchymal orchiopexy without testicular fixation sutures is inferior to conventional sutured fixation, while recurrent torsion and suture‐related complications have been documented after sutured orchiopexy.

The continued popularity of sutured fixation is likely multifactorial. Three‐point suture fixation has been practised for generations and remains quick, familiar and easily taught in the emergency setting. Testicular torsion is relatively uncommon and often presents after hours, when surgery may be performed by trainees, fellows, paediatric surgeons or general surgeons following established local protocols. Recurrent torsion or suture‐related morbidity may occur years later and may present to another institution, limiting feedback to the original surgeon or unit. Consequently, traditional suture fixation may persist because of familiarity, emergency workflow, guideline reinforcement and limited dissemination of reproducible non‐parenchymal alternatives, rather than definitive evidence of superiority.

Existing guidance also reflects ongoing uncertainty. The EAU guideline notes no consensus on the preferred fixation technique or suture material,^20^ while UK/FIX‐IT guidance continues to favour non‐absorbable sutured fixation, particularly in adults.^12^ Clearer evidence‐based guidance from professional bodies would therefore be valuable. Any recommended technique should be simple, safe and reproducible in emergency practice. Tunica vaginalis eversion and Jaboulay‐type dartos pouch techniques have previously been described with favourable outcomes,^15^, ^16^ and the present technique builds on this non‐parenchymal principle by providing a simplified, stepwise, single‐incision adult approach that reflects the tunica vaginalis proximally over the spermatic cord and avoids trans‐tunical or transparenchymal fixation sutures.

This study has several limitations. The present series represents a small, non‐comparative, single‐surgeon experience with intermediate follow‐up. Although no recurrent torsion, testicular atrophy, chronic pain or scrotal complications were observed, larger comparative studies with longer follow‐up would be valuable. However, given the low incidence of adult intermittent torsion and the rarity of recurrent torsion as an endpoint, large prospective comparative trials may be difficult to conduct. Multicentre observational studies or registry‐based evaluation may therefore be more realistic. Overall, the available experimental, clinical and consensus literature challenges the assumption that trans‐tunical suture fixation is inherently superior and supports further evaluation of dartos pouch orchiopexy as a biologically rational, non‐parenchymal alternative for selected adult patients.

## 5 CONCLUSION

Single‐incision dartos pouch orchiopexy is a simple, atraumatic and reproducible non‐parenchymal technique for adult testicular fixation. In this initial adult series, it achieved stable dependent testicular positioning with preserved vascularity and no recurrent torsion, atrophy, chronic pain or reoperation at intermediate follow‐up.

## AUTHOR CONTRIBUTIONS

**DKA:** conceptualization, data curation, methodology, writing.

## CONFLICT OF INTEREST STATEMENT

None declared.

## Data Availability

The data that support the findings of this study are available on request from the corresponding author. The data are not publicly available due to privacy or ethical restrictions.
